# Street safety through children's eyes: integrating Photovoice and machine learning to uncover disparities in environmental safety perception between children and adults

**DOI:** 10.3389/fpsyg.2025.1666290

**Published:** 2025-09-26

**Authors:** Kaiqi Wang, Chengshuai Wu, Lisha Meng, Hualong Qiu, Qinghao Zhu, Donglei Wu

**Affiliations:** ^1^Department of Art & Design, Nanjing Forestry University, Nanjing, China; ^2^School of Architecture, Central Academy of Fine Arts, Beijing, China; ^3^School of Architecture, Harbin Institute of Technology (Shenzhen), Shenzhen, China

**Keywords:** environmental safety perception, intergenerational differences, Photovoice, street view images, deep learning

## Abstract

The relationship between the built environment and human safety perception has been widely studied, but existing research lacks a child-friendly perspective in exploring the impact mechanisms of street environmental elements on children's safety perception and their intergenerational differences with adults. The study employed “Photovoice” method to assess children's and adults' perceptions of urban street safety. By integrating dual-perspective street-view images with deep learning techniques, a large-scale evaluation of street safety perception was conducted. Additionally, random forest model was used to quantify the differences in the impact of various elements on children's and adults' safety perception. Results indicate that children generally perceive lower environmental safety compared with adults, with significant differences observed in spatial preferences, attention patterns, emotional response models, and the perception of environmental elements. The study finds that vegetation, water bodies, and sidewalks positively influence children's safety perception, whereas traffic-related elements such as motor vehicles and certain complex artificial structures evoke negative reactions. Children's safety perception shows a steady trend, while adults' perception is more complex. This study provides methodological innovations and practical pathways for child-friendly urban development, emphasizing the need to consider children's unique perceptual needs and promoting a transition toward age-inclusive urban spaces.

## 1 Introduction

Against the backdrop of rapid urbanization, the proliferation of multiple and complex safety threats in urban environments (Vardoulakis and Kinney, 2019) has severely affected children's outdoor activities and their physical and mental wellbeing (Gao et al., 2024). These threats include intricate traffic conditions (Lee et al., 2018), air pollution issues (Bouazza et al., 2018), and uncertainties in the community environment (Christian et al., 2015). Such factors not only constrain children's ability to engage in free and comfortable outdoor activities (Corona, 2015) but may also induce psychological fear and anxiety toward outdoor environments, thereby diminishing their willingness to go outside (Li and Seymour, 2019). Safety, as a fundamental right of children, is a crucial element in fostering their overall development (Peck and Terry, 2021). Building a highly secure child-friendly urban environment to enhance children's perception of environmental safety is therefore of great significance for the construction of child-friendly cities and the protection of children's rights to a supportive environment (Brown et al., 2019; Gencer and Karagöz, 2017; Gill, 2021).

Children's perception of environmental safety refers to their subjective assessment of potential risk factors in a given environment, based on their cognition, experiences, and emotions, as well as the resulting psychological responses (Zeng et al., 2023). Previous research on children's perception of environmental safety has primarily focused on specific activity spaces (Senda, 2015). For instance, Ma et al. (2022) examined how to provide safe outdoor activity spaces for children in green open spaces and highlighted the important role of natural environmental factors in alleviating children's negative emotions. Similarly, Gao et al. (2024) explored how the characteristics of outdoor play areas influence children's perceived safety, revealing that the built environment and play space characteristics affect children's sense of security during activities. Zhang et al. (2023) investigated the impact of child-friendly design in urban pocket parks on children's activities, showing that safety factors within park environments positively influence children's willingness to engage in outdoor activities and their perception of safety. However, children's daily activity spaces are not limited to designated play areas (Valentine and McKendrck, 1997). The effect of street environments on children's feeling of safety has not been thoroughly investigated (Amiour et al., 2022).

As a key site for children's informal daily activities and a necessary passage for commuting to and from school (Badland et al., 2011), the layout and design of streets have a momentous effect on children's development (Bridges et al., 2020). Numerous studies on children have indicated that residential streets serve as important play spaces (Gospodini and Galani, 2006; Li and Seymour, 2019). Although these areas are not formally designed as playgrounds, their openness and flexibility make them highly attractive to children, gradually turning them into vital spaces for daily activities (Zhao et al., 2023). Existing research on children's safety in street environments primarily focuses on traffic and pedestrian safety (Deluka-Tibljaš et al., 2022; Schwebel and McClure, 2014). However, factors influencing children's psychological sense of safety should not be limited to the domain of traffic. Extensive studies on environmental safety perception have been conducted from an adult perspective. For instance, Garcia et al. found that perceived environmental safety is closely related to residents' quality of life (Garcia et al., 2024). He et al. (2023) pointed out a significant relationship between the physical environment and safety perception, where elements such as street-facing buildings, roadways, sidewalks, greenery, and street infrastructure all affect users' perception of safety. However, based on cognitive development theory, children—due to their incomplete physical and psychological development—exhibit unique physiological characteristics and heightened sensitivity (Piaget and Cook, 1952). For children in the concrete operational phase of cognitive development (aged 7–12), their thinking gradually acquires some logical structure and reversibility, enabling them to perform basic logical reasoning and categorization. However, they still require concrete objects to support their thinking and struggle with purely abstract concepts (Piaget, 2013). Under this specific cognitive pattern, tangible objects and events in the environment exert a more direct and pronounced influence on children's psychological experiences, differing from the perception models of adults, which rely more on experience and abstract reasoning (Oloumi et al., 2012). While the idea that children's perceptions and emotional responses significantly differ from those of adults has gained widespread recognition (Hyun, 2005; Ollendick et al., 1985; Silvers et al., 2012), in-depth understanding remains lacking regarding how specific environmental factors affect children's perception of safety and how these mechanisms and degrees of influence differ from those observed in adults. It is therefore imperative to explore more scientifically rigorous research methods and adopt evaluation perspectives that are more suitable for children from a child-friendly approach, to comprehensively uncover the underlying mechanisms of children's environmental safety perception.

The evaluation of children's perception of street safety should be based on their authentic experiences. Typical research methodologies such as surveys, interviews, and Virtual Reality (VR) technology, though widely used in child studies, may have certain limitations (Su and Yang, 2022). However, the “Photovoice” method offers a new perspective for child-centered research. In previous studies, Côté-Lussier et al. (2015) used surveys to explore differences in neighborhood safety perceptions between children and their parents. Guo et al. (2023) conducted interviews and field studies to examine how the environmental features of child-friendly community streets in China affect children's sense of safety. Orellana et al. (2024) examined the impact of street environments on children's perception of safety and mobility through surveys and interviews. However, survey and interview methods rely heavily on subjective perception variables and children's ability to articulate their experiences, potentially resulting in capturing only partial or biased perceptions (Segre et al., 2021). Recent years have witnessed researchers commencing the exploration of new technologies for gauging individual psychological states and perceptual preferences. Examples include the use of VR technology to simulate scenarios and track emotional changes. For instance, Shen et al. (2015) used VR to simulate children's street-crossing behavior, investigating how different emotional traits influence their risk-taking as pedestrians. However, these methods still require real-world or controlled experimental environments, making their applicability relatively narrow and limiting their generalizability (Park et al., 2019). Factors such as children's cognitive ability, comprehension skills, questionnaire format, and ease of understanding survey items can affect the accuracy of results and children's level of participation (Segre et al., 2021). As an innovative evaluation tool, the art-based research method “Photovoice” provides a novel research approach by placing cameras in children's hands and enabling them to act as community documentarians. Specifically, through the engaging artistic process of “image capture with annotation,” this method not only effectively stimulates the participation enthusiasm of children aged 7-12 and avoids information bias caused by their inattention or limited expressive ability, but also empowers children's expression from a child-centered perspective. It allows children to vividly and intuitively present first-hand views on community strengths and needs, captures details easily overlooked by adults (such as micro-environmental elements affecting children's safety, including obstruction by low shrubs and ground protrusions) based on self-captured images, compensates for the limitations of adult-led evaluations, and supports the research orientation of “being child-oriented”; meanwhile, it enables children to communicate with policymakers through visual narratives, ultimately facilitating meaningful changes (Wang and Burris, 1997). Additionally, this application approach fully respects children's agency, prompting them to express their views and experiences in a more natural and proactive manner, thereby providing richer, more authentic, and comprehensive research data (Ferraro and Maher, 2024). Notably, this study marks the first application of “Photovoice” in the field of urban environmental safety perception, particularly for the child group. It breaks through the limitation that previous similar studies mostly relied on the objective features of street view images and lacked in-depth connection with subjective perception, and provides a new paradigm for “subjective perception-objective environment” linkage analysis in subsequent research on child-friendly cities. Furthermore, integrating this innovative research method with large-scale urban street data and deep learning technologies could enable a more objective evaluation of urban environments from a child's perspective.

The use of Street-View Images (SVIs) combined with deep learning techniques for large-scale human perception analysis has been proven to be a reliable and efficient method (Yang et al., 2024), with numerous related studies in safety perception (Harvey et al., 2015; Ramírez et al., 2021). Naik et al. (2014) utilized SVIs and machine learning techniques to predict urban street safety perceptions, finding that street characteristics are closely related to perceived safety. Similarly, Hou and Chen (2024) applied this method to analyze road safety perceptions in complex urban environments, revealing that traffic infrastructure and greenery significantly impact safety perception. As research progresses, some scholars have begun applying this approach to studies on children's perceptions. Yang et al. (2024) in their study on the influence of environmental factors on children's emotions, accounted for the differences in viewing height between children and adults by simulating a child's perspective through top-cropping of SVIs obtained from mapping software. However, solely relying on image cropping presents certain limitations. First, cropping does not alter the camera angle or perspective but merely reduces the image size, which can still result in a perspective mismatch between the simulated and actual child's viewpoint. Second, children's three-dimensional perception and depth perception at lower viewing angles may differ from those of adults, a factor not fully captured in cropped images. For instance, roadside flowers, low fences, and other low-height elements can have a significant impact on children's visual perception and emotional responses. The difference in viewing height due to stature is the most fundamental physiological difference affecting how children and adults perceive their environment (Carrasco et al., 2022). Given that SVIs collected through vehicle-mounted photography systems are designed to simulate an adult pedestrian's viewpoint (Li et al., 2022), data obtained from mapping software cannot be directly applied to research on children's perspectives. To better align with children's visual perception, SVI collection should simulate how children navigate streets and replicate their eye-level perspectives. This quasi-experimental simulation method based on real-world conditions provides a technological approach for studying the visual quality of street spaces from a child pedestrian's viewpoint. Additionally, selecting an appropriate study area is crucial. As a major city in China's Yangtze River Delta region, Nanjing's urban development model is highly representative within China (Luo and Wei, 2009). The city has made notable progress in child-friendly urban initiatives, some communities have begun modifications to better accommodate children's needs (Zhu, 2024). However, Nanjing also faces challenges, particularly in older communities, where aging public infrastructure and inadequate facilities fail to meet the diverse needs of children (Shao and Liu, 2023). Suojin District, located in Nanjing's old urban district, has been designated as a national demonstration site for child-friendly community development and has made progress in establishing child-friendly environments and facilities. Yet, it also faces the common issues associated with aging communities (Yuxin, 2024). Research in this area could address challenges in child-friendly urban development, explore solutions, serve as a model for other communities, and inform nationwide child-friendly city initiatives.

On the basis of this understanding, this study aims to integrate SVIs, deep learning, and the Photovoice method to comprehensively assess and synthesize children's psychological perception of environmental safety in urban streets from a child-friendly perspective. By exploring the differences in safety perception between children and adults, the study seeks to provide practical strategies for constructing child-friendly street environments. The research aims to address the following questions: (1) What are the primary factors influencing children's perception of street environmental safety, and what are their relative weights? (2) How do children's and adults' perspectives differ in their perception of environmental safety in urban spaces?

By analyzing these questions and intergenerational perceptual differences, the study aims to propose targeted strategies to enhance safety in existing urban street environments, providing theoretical and practical guidance for child-friendly city development, ensuring children's right to safety, and advancing sustainable urban and social harmony.

## 2 Materials and methods

### 2.1 Study area

**Figure 1:**
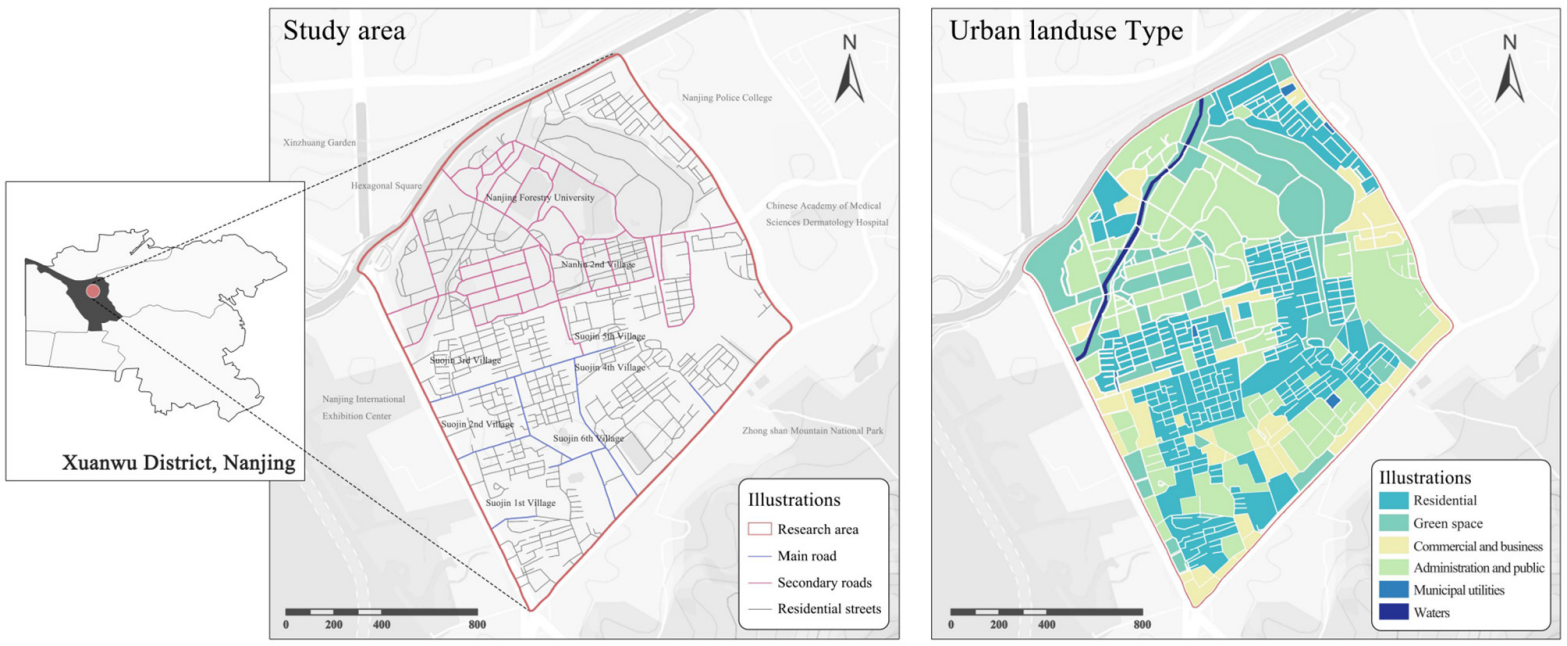
Study scope and urban land use type.

The study area is situated in the core of Suojin District, Xuanwu District, Nanjing, covering approximately 196.6 hectares. Spatially, it is centered around Nanjing Forestry University and Suojin Village, with clearly defined boundaries, as shown in Figure 1. As a designated national demonstration site for child-friendly community development, this district has a high concentration of primary education resources, including multiple high-quality schools. It also encompasses more than 10 residential communities and university-affiliated housing areas, forming a high-density child population cluster. However, the spatial environment of this area exhibits a complex and multi-layered structure, comprising static spaces such as university teaching areas and old residential complexes, while also being adjacent to major urban traffic arteries like Longpan Road and Xuanwu Avenue. These conditions contribute to fragmented public spaces and the interweaving of pedestrian and vehicular traffic flows, creating a complex urban environment (Yuxin, 2024), makes it a valuable case study for optimizing child-friendly urban spaces.

### 2.2 Research framework and methods

**Figure 2:**
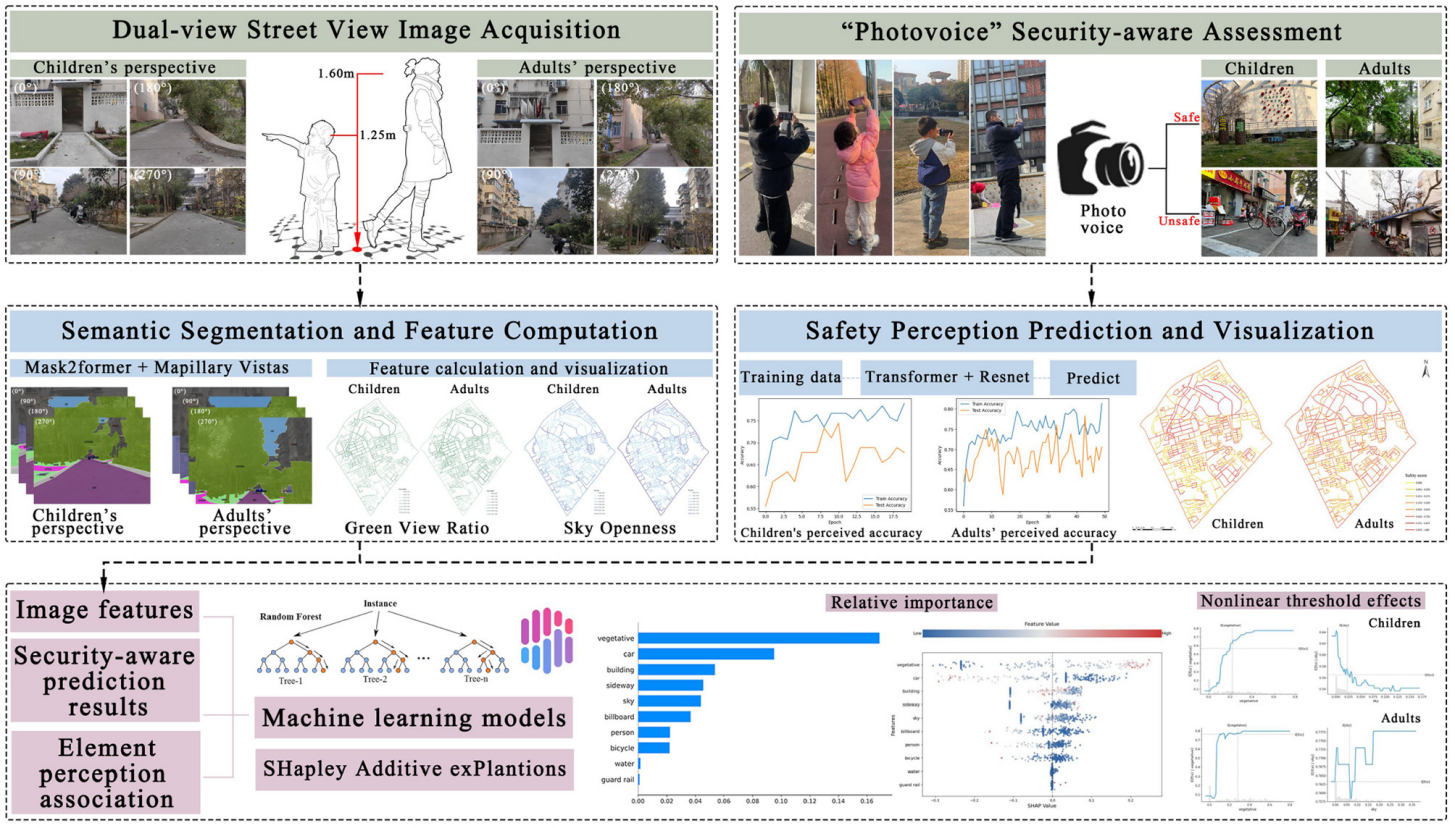
Analytical framework.

This study focuses on the perceptual evaluation of urban streetscapes from a dual-perspective approach—child and adult viewpoints—integrating perception assessment with planning decision-making, as illustrated in Figure 2. Firstly, the dual-view SVIs of children and adults in the study area were collected by artificial shooting, and the binary labels of safety perception were labeled by using the “Photovoice” method. Next, a semantic segmentation model is introduced to analyze the environmental components in the street-view images, extracting the spatial distribution characteristics of elements. A binary safety perception predictor is constructed to simulate perception across the entire streetscape environment. A non-linear regression model is finally employed to quantify the influence weights of different environmental factors on safety perception, enabling a comparative analysis of differences between adult and child perception. This process forms a closed-loop process from data collection and model analysis to decision-making support, ensuring an evidence-based approach to improving urban streetscapes for children.

Street-View Imagery (SVI) is a crucial data source in this study. The research utilizes the OpenStreetMap (https://www.openstreetmap.org/) open-source platform to obtain road network data for the Suojin District in Nanjing. A total of two thousand two hundred and sixty nine street-view sampling points were evenly spaced at 20 m intervals across the study area. SVIs were taken at the sampling points to collect different visual heights for children and adults. This study recruited six volunteers to participate in the capture of dual-perspective SVIs, with the shooting scope covering the entire study area. SVIs were taken at the sampling points to collect different visual heights for children and adults. For the collection of child-perspective SVI, the study aimed to accurately capture children's field of vision by simulating their movement and eye-level perspective along the streets. According to the 2024 Children's Height Status Report (Fund, 2024), the height range for children aged 7–12 is 124 cm to 152 cm, with an average eye height of 110 cm to 140 cm. In order to ensure that the SVI is as close as possible to the real field of view of children, the simulated shooting angle is selected between 125 cm and 130 cm. A total of 8,776 SVIs were collected during the sunny day in December 2024, excluding spaces that could not be collected due to closed management. At the same time, in order to ensure that the spatial orientation of the images is consistent with that of children's perspectives, the SVIs from the perspective of adults are also self-collected, this study selected the average eye height of Chinese adults, 160 cm, as the shooting height, and finally 8,776 images with the same sampling point orientation and the same time as the children's perspective are collected. After collecting all SVIs, the datasets were processed separately for adult and child perspectives, forming a foundation for semantic segmentation and machine-simulated perception analysis.

To achieve precise fine-grained recognition and classification of scene elements, this study employs the advanced panoptic segmentation framework Mask2Former for semantic segmentation, while utilizing the Mapillary Vistas dataset for model training and optimization. Mask2Former, an advanced Transformer-based semantic segmentation model (Cheng et al., 2022), has demonstrated distinct advantages in semantic segmentation tasks using the Mapillary Vistas dataset. The Mapillary Vistas dataset is an extensive image resource repository containing a wide variety of SVIs. It covers typical urban scene elements, including roads, buildings, pedestrians, with finely annotated labels spanning 66 different categories. This dataset provides a solid data foundation for model training and optimization (Neuhold et al., 2017). When processing the Mapillary Vistas dataset, Mask2Former leverages the powerful global modeling capabilities of Transformers to efficiently capture and deeply integrate contextual information within images. This approach allows the model to accurately segment individual target objects in complex and dynamic urban street scenes, significantly improving the accuracy and robustness of semantic segmentation results (Cheng et al., 2022).

Finally, the collected SVIs were subjected to semantic segmentation, allowing for the precise calculation of the proportion of each environmental element within the images. Considering well-documented characteristics of children's cognition, such as their tendency for distractibility (Rueda et al., 2004), weaker ability to anticipate danger (Meyer et al., 2014), and behavioral patterns, including limited activity range (Fisher et al., 2015) and randomness in movement (Gong and He, 2016), the selection of evaluation variables prioritized those most likely to influence children's perception of safety. Specifically, 10 environmental elements were chosen, as shown in Table 1. The study further analyzed and explained the bidirectional relationship between these elements and safety perception.

**Table 1:** Descriptions of the street environment metrics used in this study.

**Metrics**	**Descriptions of metrics and complementary explanation**	**Adults result**		**Child result**	
		**Mean**	**SD**	**Mean**	**SD**
**Natural**					
Vegetation	Visible proportion of greenery in the street view. Moderate vegetation provides a sense of security and natural affinity, but excessive density may obstruct visibility, increasing potential risks.	0.275	0.046	0.202	0.028
Sky	Proportion of sky area unobstructed by buildings or facilities.A clear sky can create an open and spacious feeling, reducing feelings of confinement.	0.066	0.007	0.023	0.001
Water	Visible proportion of natural or artificial water bodies. Water bodies add to the aesthetic appeal and can create a calming environment.	0.001	0.000	0.000	0.000
**Infrastructure**					
Buildings	Visual proportion and density of building and wall. High-density buildings may compress activity space and create oppression, while low-rise buildings with rational layouts can offer refuge.	0.199	0.029	0.183	0.023
Sidewalks	The proportion of paved surfaces. Wide and continuous sidewalks ensure safe walking for children; damaged or narrow sidewalks increase fall risks or force children into vehicle lanes.	0.020	0.001	0.031	0.001
Billboard	Proportion of billboards and advertising signs. Billboards can be visually distracting and may reduce the natural aesthetic.	0.007	0.000	0.007	0.000
Guardrails	Presence ratio of protective guardrails. Guardrails create boundaries and limit access, affecting children's sense of freedom and safety.	0.000	0.000	0.000	0.000
**Traffic**					
Motor vehicles	The proportion of motor vehicles. High traffic volume can create a sense of danger and noise, reducing children's feelings of safety.	0.035	0.002	0.043	0.004
Non-motorized vehicles	The proportion of non-motorized vehicles. Non-motorized vehicles can create a sense of activity and movement without the danger of motorized traffic.	0.004	0.000	0.007	0.000
Pedestrian	Proportion of pedestrians in the image. The presence of pedestrians can create a sense of community and social interaction.	0.002	0.000	0.002	0.000

For the construction of the human-machine adversarial perception prediction model, this study adopted innovative research method “Photovoice” for children and adults. To ensure sample representativeness, participants were recruited via a “community collaboration with stratified sampling” strategy (demographic details in Table 2): thirty children from 3 local public primary schools, and 30 adults with the assistance of 10 community neighborhood committees. The Photovoice implementation involved: brief training for participants to clarify objectives (documenting “safe/dangerous” environments to reflect safety perception levels) and shooting rules; a 7-day independent image capture period covering all spatial types in the study area, with participants providing short explanations for their judgments; and independent screening by two researchers to exclude invalid samples. Ultimately, eight hundred and sixty seven valid safety perception photos from each group were obtained, which were used to build the safety perception labeling system for subsequent model training. This research strictly adheres to ethical guidelines. It involves no human experimentation, having obtained ethical approval from Nanjing Forestry University. For child participants, verbal informed consent was first acquired from the children themselves, and written informed consent was formally secured from their guardians, who signed the informed—consent forms. All findings are used solely for academic purposes, with no commercial utilization whatsoever. Moreover, as no facial information exists in the photos, data privacy is inherently protected through the absence of personally identifiable visual elements, further ensuring participants' information security.

**Table 2:** Demographic characteristics of study participants.

**Characteristic**	**Children (*n* = 30)**	**Adults (*n* = 30)**
Age range	7–12 years	18–45 years
Age distribution	7–8 years: 8; 9–10 years: 10; 11–12 years: 12	18–25 years: 12; 26–35 years: 12; 36–45 years: 6
Gender(*n*, %)	Male: 16(53.3%); Female: 14(46.7%)	Male: 15(50.0%); Female: 15(50.0%)
Height range	120–150 cm	150–180 cm
Residential status	Local household registration, living in the study area for ≥1 year	Local household registration, living in the study area for ≥1 year
Recruitment source	3 public primary schools in the study area (Suojin Xincun No.1 Primary School, Branch of Beijing East Road Primary School, Yinghua Primary School)	10 community committees in the study area (including Suojin Community No.1 to No.10)

To accurately and efficiently assess the safety perception of the built environment on a large scale, this study developed a human-machine adversarial prediction model, and considering the limited sample size, it adopted a hybrid architecture that integrates a pre-trained ResNet-152 backbone network with a Transformer module for perception modeling and optimization tailored to both children and adults; the ResNet-152 model, pre-trained on ImageNet, serves as a robust feature extractor capable of capturing hierarchical visual representations with strong cross-domain generalization ability (He et al., 2016), and given that children and adults tend to focus on different aspects of micro-level elements and macro-level scenes due to differences in eye level, ResNet's residual structure was specifically selected to effectively mitigate the degradation problem in deep networks while capturing multi-level convolutional features—ranging from edge textures to object-level local information; subsequently, the Transformer module leverages its self-attention mechanism to model long-distance contextual dependencies within the extracted feature sequences (Vaswani et al., 2017), and this combination of ResNet and Transformer enables the model to characterize both micro-element saliency and macro-scene semantics (Shaheed et al., 2023), thereby enhancing its alignment with the actual perceptual experiences of children and adults; to alleviate the constraints imposed by the small dataset scale—with 867 images per group collected via a photo selector—comprehensive data augmentation techniques (including random cropping, color jitter, and rotation) were employed, and transfer learning was further utilized to fine-tune the model, ensuring that even with limited sample size, the model can still achieve effective learning and competitive prediction performance in perception-related tasks.

For model training, 70% of labeled safety perception images from adults and children were used, with 30% reserved for testing. The adult model achieved 80% test accuracy, showing effectiveness in capturing adult perceptions. The child model had 75% accuracy, which mainly reflects the greater individual differences in children's safety judgments—their perceptions are more influenced by personal experiences and preferences—while also confirming the effectiveness of the Photovoice method in capturing authentic child perceptions; overall, the model demonstrated strong predictive capabilities, laying a reliable foundation for subsequent analysis of children's environmental safety perception characteristics. The trained ResNet hybrid prediction model was applied to all sampled points within the study area to predict safety perception scores separately for children and adults based on their respective street-view images. An independent *t-*test was conducted to examine the statistical differences in safety perception between the two groups, as shown in Table 3. The results indicate a significant difference in safety perception between adults and children. To further identify high-risk areas and optimize resource allocation, the predicted perception scores were spatially visualized, and a safety perception score map was generated.

**Table 3:** Independent-sample *t-*test.

**Indicator**	**Group (M** ±**SD)**		***t***	***p***
	**Adult (*****n*** = **8,776)**	**Child (*****n*** = **8,776)**		
Security forecast	0.66 ± 0.48	0.59 ± 0.49	8.473	0.000^**^

^**^ *p* < 0.01.

**Figure 3:**
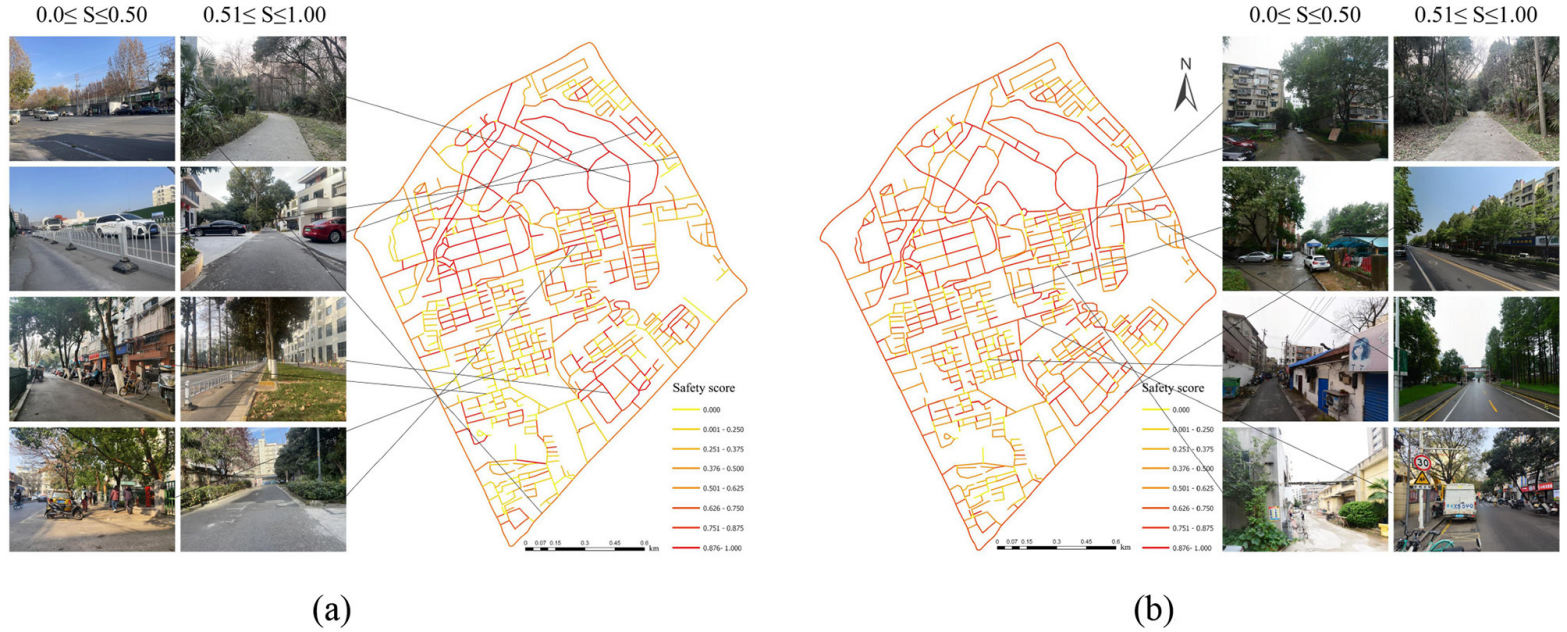
Environmental safety perception map and SVI examples. **(a)** Child **(b)** Adult.

Figure 3 presents the spatial distribution of safety perception and SVI examples for children and adults within the study area. Overall, children exhibit lower perceived environmental safety than adults. High safety perception areas for children are mainly within internal spaces of various land-use types, while adults tend to perceive internal complex road environments as more hazardous. In contrast, adults feel safer in open-view road environments and commercial areas along residential peripheries. Comparative analysis across land-use types reveals that both groups have similar high safety perceptions in green spaces and public management service areas. However, children's safety perception is significantly lower than adults' on urban arterial roads at the study area's periphery. In residential areas, children perceive higher safety within communities than adults, while in commercial areas along the region's outer edges and residential peripheries, adults feel safer than children. These findings suggest children are more inclined to perceive higher safety within internal spaces of various land-use types rather than in open or transitional environments compared to adults.

### 2.3 Random forest regression analysis

**Figure 4:**
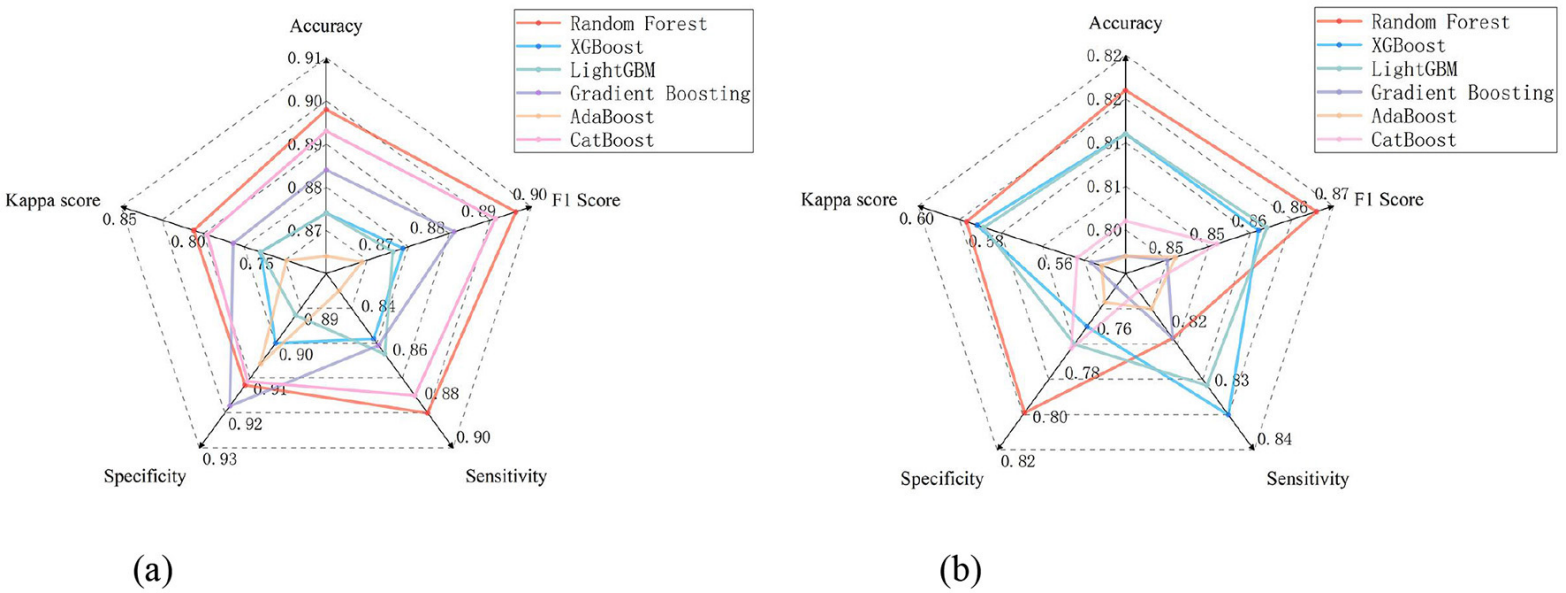
Radar Plots Comparing Model Performances for Children's and Adults'Safety Perception Modeling. **(a)** Modeling Data for Children **(b)** Modeling Data for Adults.

Decision-tree-based models, such as Random Forest, XGBoost, LightGBM, Gradient Boosting, AdaBoost and CatBoost, have become prevalent in machine-learning-driven environmental perception research. These models leverage the hierarchical decision-making structure of trees to handle complex, non-linear relationships between environmental factors and perceptual outcomes, with each variant featuring distinct optimization strategies. Given their widespread application in capturing streetscape-human perception interactions, a comparative analysis of these popular decision-tree type models is essential to identify the most suitable approach for safety perception quantification. In this study, six models were evaluated using five metrics—Accuracy, Kappa score, Specificity, Sensitivity, and F1 Score—with their performance visualized in the radar plot (Figure 4). The comparative results indicate that Random Forest outperformed the other models across all evaluated metrics. Specifically, it achieved the optimal performance in both the child safety perception model and the adult safety perception model, as evidenced by its highest Accuracy and balanced performance across all indicators. Consequently, Random Forest was selected as the final model for quantifying safety perception in this study.

**Figure 5:**
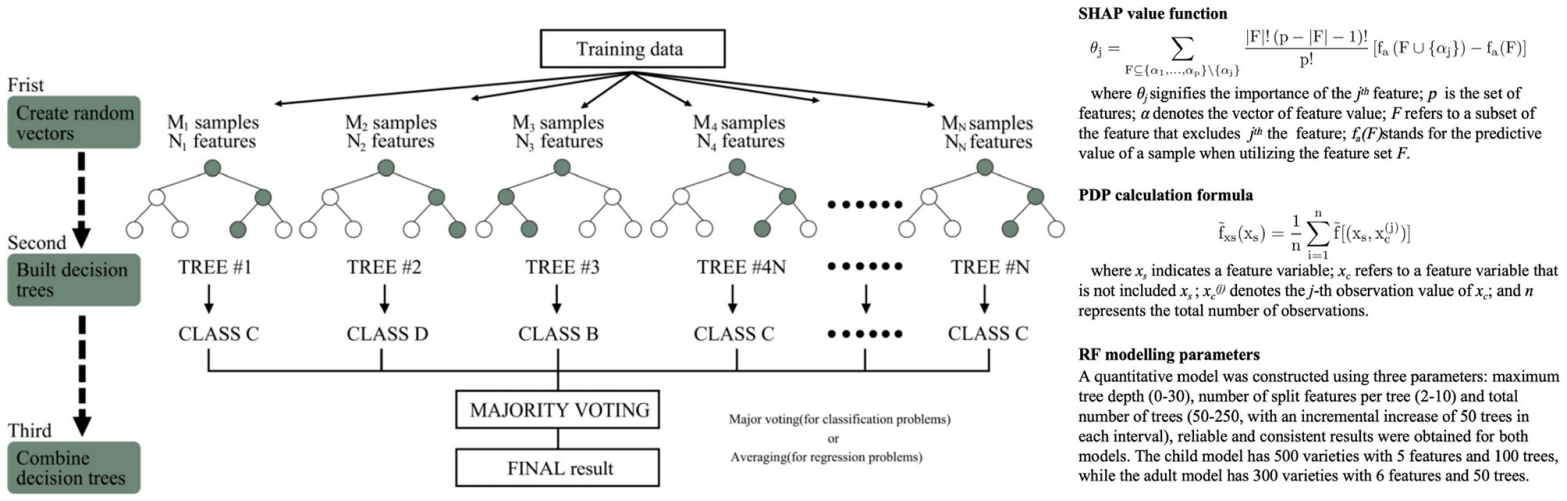
Random forest technique example and calculation methods of SHAP and PDP.

As an advanced ensemble learning method, the random forest regression model demonstrates unique advantages in capturing the complex relationships between streetscape environmental elements and human perception. It effectively models non-linear interactions between environmental features and human perception by integrating a multiple-decision-tree prediction mechanism with feature interpretation, thereby overcoming the limitations of traditional analytical methods (Breiman, 2001). The process flow of this method is depicted in Figure 5. This study used three parameters to build quantitative models for children's and adults' safety perception. The children's model used 500 samples, 5 features, and 100 trees, while the adult model used 300 samples, 6 features, and 50 trees. Results in Table 4 show strong model performance, confirming the feasibility of using random forest regression to quantify safety perception.

**Table 4:** The sample size and RF-accuracy of children and adults.

**Population groups**	**Sample size**	**Accuracy**	**F1 score**	**Specificity**	**Sensitivity**	**Kappa score**
Children	*n* = 8776	0.898	0.897	0.912	0.884	0.797
Adults	*n* = 8776	0.816	0.863	0.803	0.821	0.586

In addition, considering the specific need for model interpretability in environmental safety perception research, Shapley additive explanations (SHAP) values were introduced to analyze the marginal contribution of each environmental factor. This approach quantifies the differentiated impact of various street elements on children's and adults' safety perception and reveals the complex interaction effects between features (Iban, 2022; Wojtuch et al., 2021). Furthermore, Partial Dependence Plot (PDP) analysis was employed to visualize the functional relationship between individual environmental elements and perception scores while controlling for other variables. This method effectively distinguishes between linear and non-linear interaction patterns (Gao et al., 2023). The computational methods for SHAP and PDP are illustrated in Figure 5.

## 3 Results

### 3.1 Influence magnitude and relative importance of environmental factors

**Figure 6:**
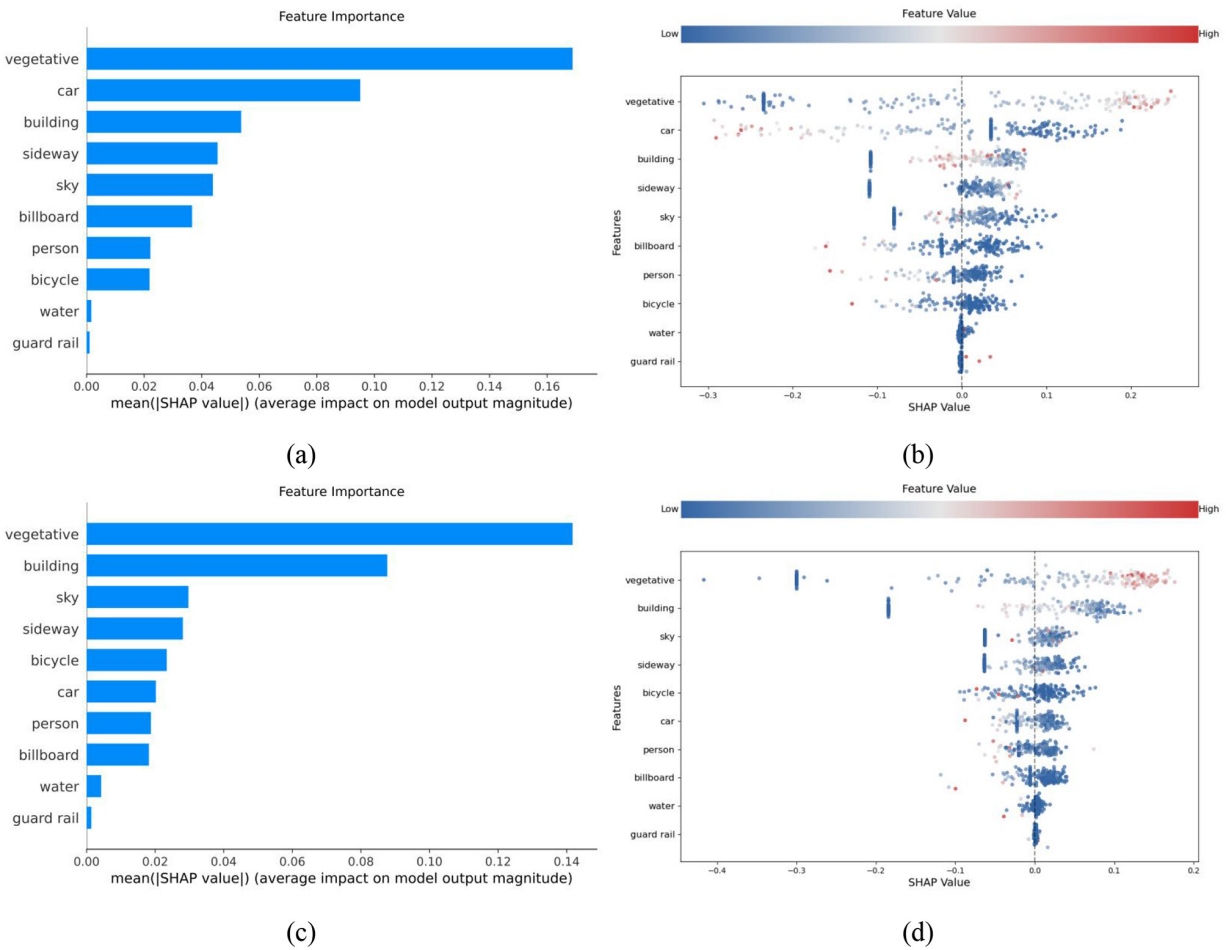
Order of significance and bee swarm plots for impact of environmental elements. **(a, b)** Child safety perception, **(c, d)** Adult safety perception.

This study employed a random forest model combined with the SHAP algorithm to explore the relationships and differences between streetscape elements and the safety perception of children and adults. Figure 6 presents the importance ranking and swarm plots of environmental factors associated with safety perception for both groups. The importance ranking diagram clearly displays the order of significance for each environmental element in influencing children's and adults' safety perception. The swarm plot provides an intuitive visualization of the distribution and dispersion of SHAP values for each feature in the model. This approach effectively illustrates the relative impact of different factors on safety perception (Iban, 2022).

The results of the importance ranking and swarm plots indicate significant heterogeneity in the influence of different environmental factors on children's and adults' safety perception. As shown in Figure 6a, the five most influential factors on children's safety perception are vegetation, motor vehicles, buildings, sidewalks, and the sky. When this result is combined with the analysis in Figure 6b, children's safety perception is evidently highly affected by environmental elements, with vegetation, motor vehicles, and buildings having the greatest impact. By contrast, Figure 6c shows that the five factors with the highest impact on adult safety perception are vegetation, buildings, the sky, sidewalks, and bicycles. According to Figure 6d, only vegetation and buildings have a significant effect on adults' safety perception, while the influence of other factors is relatively minor. Overall, vegetation consistently exerts the most significant impact on children's and adults' safety perception models. However, the influence of motor vehicles, buildings, billboards, the sky, and bicycles differs significantly between the two groups. This discrepancy aligns with differences in environmental cognition between children and adults, reflecting their distinct perceptual needs and areas of focus in evaluating safety within the urban environment.

### 3.2 Impact of environmental factors on children's and adults' safety perception

**Figure 7:**
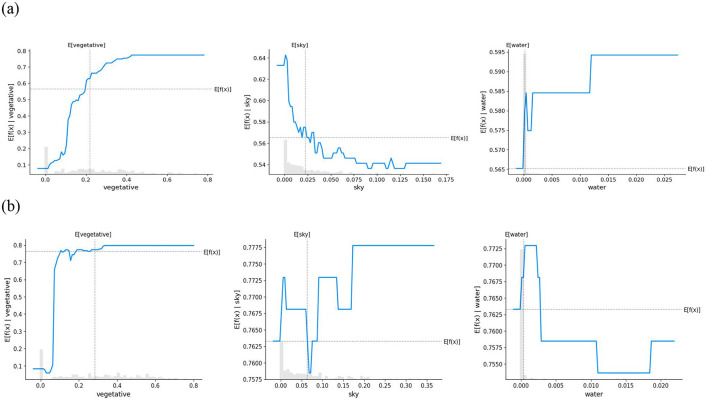
PDPs for the effect of natural elemental features on security perceptions. **(a)** Child **(b)** Adult.

**Figure 8:**
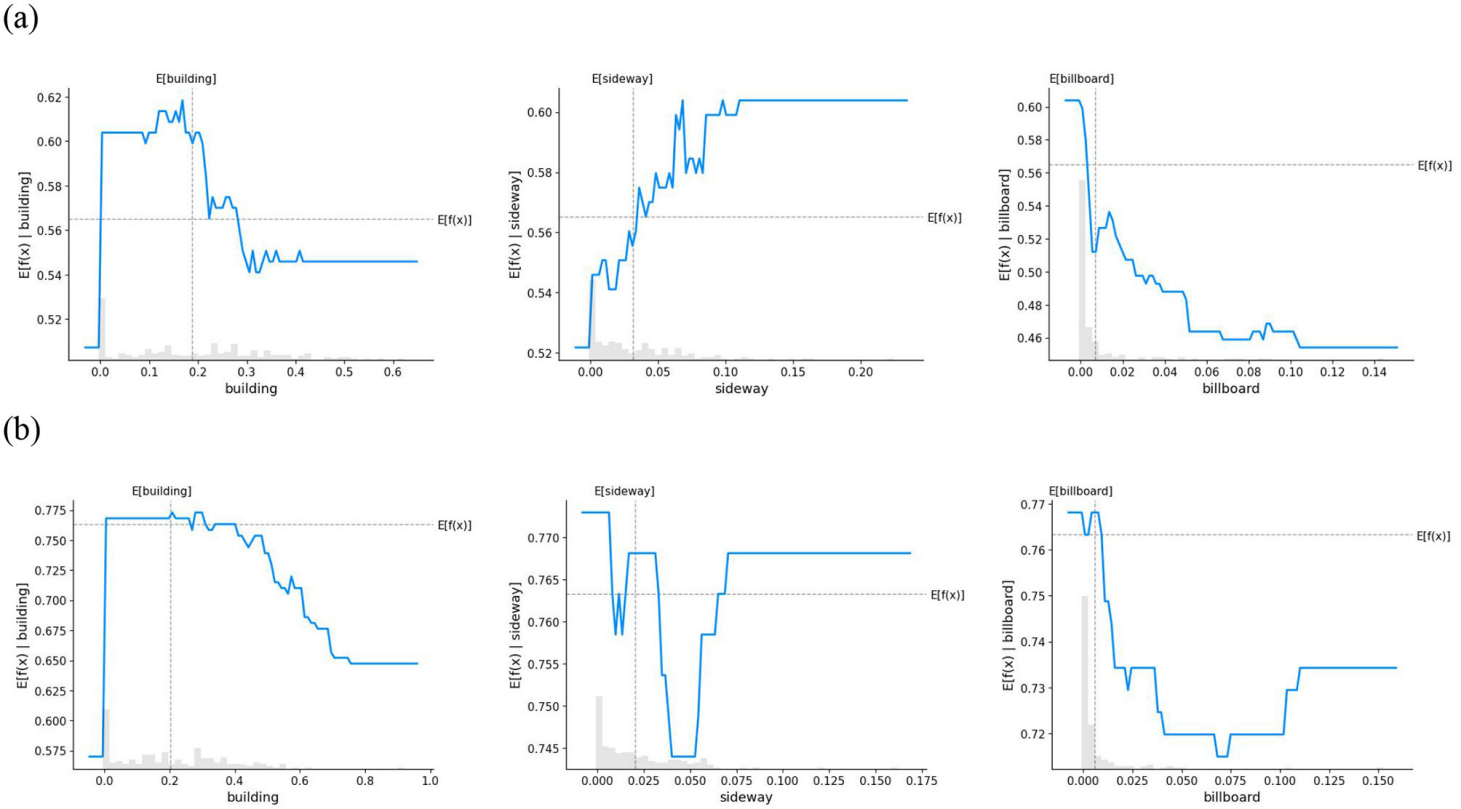
PDPs for the effect of artificial elemental features on security perceptions. **(a)** Child **(b)** Adult.

**Figure 9:**
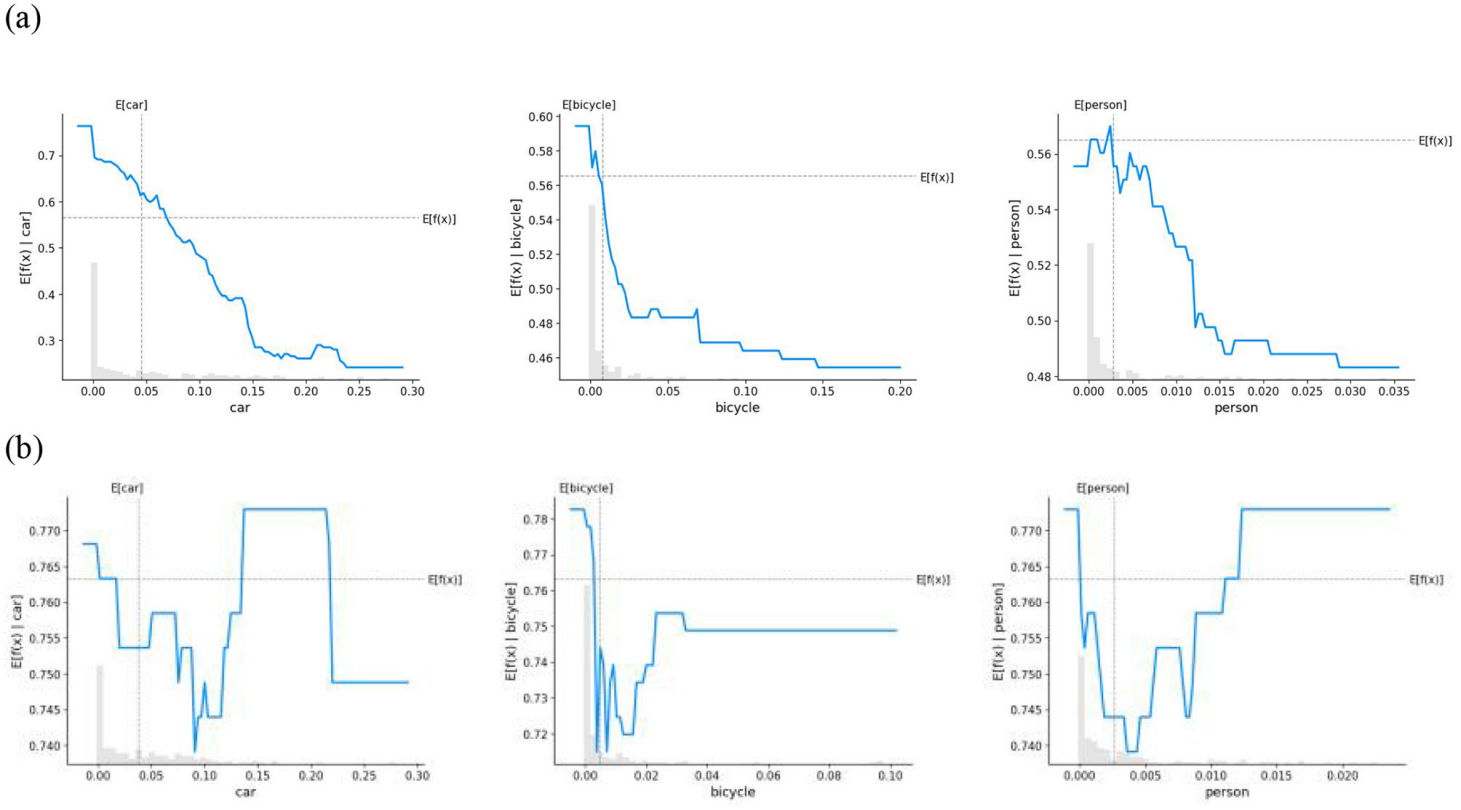
PDPs for the effect of transport elemental features on security perceptions. **(a)** Child **(b)** Adult.

To further clarify the differences in how various environmental factors influence safety perception in children and adults, this study utilized PDPs combined with stacked histograms for visualization, providing a more comprehensive representation of the impact of features on model predictions. Figures 7–9 illustrate the non-linear relationships between different environmental elements and the safety perception of children and adults.

Figure 7 illustrates the impact patterns of natural features on the safety perception of children and adults. Vegetation coverage exhibits a positive effect on the safety perception of both groups, and this positive influence is more pronounced particularly when the coverage level is low. In contrast, the impact of sky exposure on the two groups shows significant heterogeneity. For children, higher sky exposure is generally associated with a decline in safety perception: their perception score peaks at 0.64 under low sky exposure, then decreases sharply, and stabilizes at 0.54 when the sky proportion reaches approximately 0.1. Adults, however, demonstrate a more complex non-linear response: their safety perception first decreases from 0 to 0.7575 within the sky exposure range of 0.05, then gradually rebounds, and finally stabilizes at 0.7775. Regarding the water body factor, children's safety perception generally increases with the rise in water body proportion. Adults, on the other hand, exhibit an obvious threshold effect: their safety perception peaks at 0.7725 under low water body exposure, but begins to show a downward trend when the water body coverage exceeds 0.003, followed by a slight recovery and eventual stabilization at 0.02.

Figure 8 clarifies the impact of infrastructure elements on the safety perception of children and adults. Given that the impact of guardrails is weak and statistically insignificant, guardrails were excluded from this analysis. For buildings, children's safety perception presents a significant threshold effect: their safety perception is positively correlated with building elements until the proportion reaches 0.2 (at which point the perception score peaks at 0.62), then drops sharply, and stabilizes at 0.54 when the proportion approaches 0.4. For adults, safety perception decreases linearly with the increase in building density and stabilizes when the proportion reaches 0.8. The impact of sidewalks also differs between the two groups: children's safety perception increases with the growth of sidewalk elements. In comparison, adults exhibit non-linear characteristics: when the sidewalk proportion ranges from 0 to 0.05, there is a significant negative impact on safety perception; however, after 0.05, safety perception increases significantly and stabilizes at 0.77 when the proportion reaches approximately 0.075. Billboards generally exert a negative impact on children's safety perception. For adults, nevertheless, the impact of billboards shows a non-linear effect: when the proportion ranges from 0 to 0.075, billboards are negatively correlated with safety perception, but after the perception score reaches the minimum value of approximately 0.7, this correlation shifts to a positive one.

Figure 9 presents the impact of traffic elements on the safety perception of children and adults. For children, both motor vehicles and non-motor vehicles have a negative impact on safety perception. Pedestrian elements exert a positive impact on safety perception when their proportion is below 0.005; however, once this threshold is exceeded, the perception degree decreases significantly and stabilizes at 0.48 when the proportion reaches approximately 0.015. For adults, all three traffic factors exhibit complex non-linear relationships. When the proportion of motor vehicles is below 0.1, it is negatively correlated with safety perception (with the minimum predicted value being 0.74), then rebounds and fluctuates, and finally stabilizes at approximately 0.75. Non-motor vehicles lead to a sharp decline in safety perception, especially when the bicycle density ranges from 0 to 0.02; after that, the perception degree gradually recovers to around 0.75. Pedestrian density has a negative impact on safety perception when it is below 0.005, but after this point, the perception degree rises and peaks at 0.77.

## 4 Discussion

### 4.1 Differences in safety perception patterns between children and adults

Through learning, simulation, and quantitative analysis of children's and adults' safety perception assessments, this study identified significant differences in their spatial preferences, attention patterns, and emotional responses to the environment. First, compared to adults, children generally have lower environmental safety perception. Both groups show similar high safety perception only in green spaces and public service areas. Children feel safer in residential areas, while adults feel more secure in commercial zones near residential areas and open traffic corridors. This finding highlights children's preference for enclosed or internal spaces, which may be attributed to their familiarity with residential environments (Hume et al., 2005) and the lack of consideration for children's needs in urban planning. Many planning decisions prioritize transportation and commercial development (Yang et al., 2024), resulting in a shortage of child-friendly infrastructure, which is often limited to residential areas (Katz and McLeigh, 2017). These results indicate an urgent need to improve the child-friendliness of urban streets. Notably, this spatial perception difference also aligns with the accuracy gap between the child safety perception model (75%) and the adult model (80%): children's judgments of environmental safety are more susceptible to personal experiences and preferences, resulting in lower consistency in perceptions among individuals, which has affected the model's fitting degree to a certain extent. In contrast, adults' perceptions rely more on objective and consistent risk cues, leading to higher model accuracy.

Second, the importance ranking of safety perception factors and the swarm plot analysis reveal differences in the focus patterns of children and adults regarding safety perception. Children's safety perception relies more on direct sensory stimuli, while adults base theirs on comprehensive environmental evaluation and experience. For children, six key factors significantly impact their psychological safety perception: vegetation, motor vehicles, buildings, sidewalks, the sky, and billboards. These elements are prominently featured in their visual field. Conversely, for adults, only vegetation and buildings strongly influence safety perception, with other elements having smaller, more evenly distributed impacts. This may relate to adults' greater environmental adaptability and preference for stability and order. According to person–environment fit theory, environmental stability and order can significantly enhance adults' adaptability and reduce stress (Kristof-Brown et al., 2005). However, from the perspective of child developmental psychology, children's attention is highly selective and easily shifted, and their cognitive and judgment abilities are relatively underdeveloped (Hagen and Hale, 1973; Piaget and Cook, 1952). As a result, children are more susceptible to direct environmental influences, potentially explaining why their safety perception is more strongly shaped by prominent environmental features.

From the overall trend of the partial dependence plot curves depicting the relationship between environmental elements and safety perception, distinct differences in emotional response patterns between children and adults were observed. Children's safety perception tends to exhibit a relatively stable linear pattern of change, by contrast, adults' safety perception is characterized by complex non-linear variations. The complex non-linear relationship between adults' emotions and environmental factors has been widely documented in previous studies (Cui et al., 2023; Xu et al., 2023). This phenomenon is often attributed to their fully developed physiological and psychological functions, as well as social and contextual influences (Ghosh and Halder, 2020). Compared with adults, children aged 7 to 12 are still in the developmental stage of cognitive abilities. Although they are beginning to understand logical concepts, they still struggle with abstract reasoning and complex analysis (Piaget and Cook, 1952). As a result, their perception and interpretation of the environment tend to be simpler and more direct, leading to a more linear or straightforward pattern in safety perception. Furthermore, children's limited information-processing capacity makes them more prone to focusing on immediate and prominent stimuli, while overlooking other potential safety-related factors. This constrained ability to process information prevents children from conducting a comprehensive analysis of complex environments, resulting in simpler and more direct safety perceptions (Gaspelin et al., 2015). Overall, children's safety perception is more directly influenced by environmental factors and follows a linear pattern, while adults' perception, based on experience-based comprehensive assessments, shows non-linear fluctuations. These findings are linked to children's cognitive development stage and the lack of child-friendly facilities in urban planning.

### 4.2 Differences in the impact of environmental features on safety perception

From the perspective of environmental features, the study found that children and adults show similar patterns in safety perception regarding vegetation and buildings. Both groups perceive increased safety with higher vegetation coverage, as vegetation provides shade and visual comfort, enhancing the psychological sense of security. This finding aligns with numerous studies on green visibility, such as Ward et al., who explored the impact of children's exposure to green spaces on their physical activity, cognitive development, emotional wellbeing, and risk assessment abilities. Their findings confirmed that green space exposure positively affects children's emotional health (Ward et al., 2016). Similarly, Sezavar demonstrated that well-planned vegetation design can significantly enhance adults' sense of safety and comfort in parks (Sezavar et al., 2023). Regarding building elements, the results show that low-density built environments positively influence safety perception for both children and adults, likely due to more open spaces and activity areas, as well as a sense of enclosure and spatial boundaries. However, high-density built environments have a negative impact on the psychological sense of safety for both groups. This conclusion is consistent with findings from Zhang and Maas, who demonstrated that high-density built environments negatively correlate with residents' sense of security and overall health status (Maas et al., 2006; Zhang et al., 2018). These findings highlight the importance of balancing building density and green space proportion in urban planning to optimize safety perception and wellbeing.

However, the perception of other environmental elements differs significantly between children and adults. Among these, water bodies and sidewalks exhibit a positive correlation with children's safety perception, whereas adults display a threshold effect in response to these elements. Children's safety perception of water bodies is largely influenced by their natural curiosity and cognitive development. They are instinctively drawn to water and enjoy exploring it because it fulfills an innate desire for sensory engagement (Gross, 2012). However, their awareness of drowning risks is weak, and they lack the rational thinking ability to assess such dangers. As Joshi et al. pointed out, children tend to demonstrate unrealistic optimism regarding drowning risks, believing that they are unlikely to encounter such incidents and underestimating the probability of accidents (Joshi et al., 2018). By contrast, adults have a more developed risk assessment ability and psychological flexibility (Kashdan and Rottenberg, 2010). When water bodies are limited in number, adults generally find them aesthetically pleasing and beneficial for environmental comfort, enhancing their sense of safety. However, as the presence of water bodies exceeds a certain threshold, safety concerns arise, leading to a decline in perceived security. Regarding sidewalks, findings from Amiour et al. support the conclusion that sidewalks play a crucial role in reducing children's collision risks, aligning with this study's findings (Amiour et al., 2022). Adequate sidewalk space enhances children's safety perception by providing safe walking and play areas, reducing exposure to motor vehicles, and visibly meeting their mobility needs. For adults, increasing sidewalk availability improves safety perception until a threshold is reached, beyond which excessive space may cause discomfort, complexity, and non-motorized vehicle encroachment, limiting further benefits. Research by Hu et al. demonstrated that the absence of sidewalks at street intersections correlates with higher accident severity, while the presence of sidewalks on both sides of the road is associated with increased accident frequency (Hu et al., 2024). This finding aligns with the results of this study from the adult perspective, suggesting that while sidewalks contribute to pedestrian safety, they may also introduce new risks and potential hazards.

Research indicates that an increase in traffic-related elements negatively impacts children's safety perception. Numerous studies on children's traffic safety have demonstrated that complex traffic environments, particularly the increased presence of various traffic participants, reduce children's sense of safety (Amiour et al., 2022; Macpherson et al., 1998), aligning with the findings of this study. Several factors contribute to this effect. From a cognitive and behavioral perspective, children at certain developmental stages have limited ability to assess and respond to complex environments (Piaget, 2013). The presence of more traffic participants increases environmental complexity and unpredictability, making it difficult for children to anticipate dangers and avoid potential conflicts, thereby reducing their perceived safety. From an environmental psychology perspective, the noise and congestion caused by high traffic density create psychological stress, significantly disrupting children's ability to assess safety, further diminishing their sense of security (Bina et al., 2021; Crombie et al., 2011). In contrast, adults generally exhibit a more complex emotional response to traffic elements in terms of safety perception, which can be explained by psychological flexibility (Kashdan and Rottenberg, 2010). When traffic volume is low, vehicles tend to travel at high speeds, potentially heightening safety concerns due to the increased risk associated with fast-moving traffic. However, as traffic volume increases, vehicle speed often decreases, which can, to some extent, reduce accident risks and improve perceived safety. Nevertheless, when traffic congestion reaches a critical level, excessive crowding and disorder may once again lead to a decline in safety perception.

The elements of sky and billboards also exhibit an overall negative correlation with children's safety perception, whereas adults show an initial decline followed by an increase in perception. The differences in attention and focus between children and adults can effectively explain this phenomenon. Children's attention is more easily drawn to novel and engaging stimuli (Takacs and Bus, 2016). Unusual visual features in the sky or the bright colors and animated content on billboards can easily distract them, causing them to overlook potential dangers in their surroundings and leading to a decrease in safety perception. While adults may also be momentarily attracted to billboards, they are better at allocating attention and can simultaneously focus on these elements while maintaining an awareness of environmental safety (Kashdan and Rottenberg, 2010). Moreover, as exposure to sky and billboard elements increases, adults gradually adapt and are able to redirect their focus toward ensuring their safety, resulting in an eventual increase in safety perception. Overall, while children and adults show similar safety perception patterns regarding vegetation and building, significant differences exist in other factors. Urban planning should balance building density and green spaces, and consider the distinct perceptions of environmental factors between the two groups.

### 4.3 New directions for enhancing safety in child-friendly cities

For the development and optimization of child-friendly cities, the key priority is to ensure that urban spatial planning fully considers children's safety needs (Krishnamurthy, 2019). Given that children feel relatively safer within residential areas but less secure in external urban environments, a possible strategy is to establish a “child-friendly street life circle” by extending the safety-focused planning outward from residential areas within a radius of approximately 200 m. Within this designated area, road functions and layouts should be optimized to seamlessly connect residential zones with nearby commercial areas and public service facilities. Additionally, small green spaces and recreational areas should be integrated along streets within this life circle. Thoughtful placement of sandpits, climbing structures, and other children's play facilities can help extend children's activity spaces beyond residential areas, creating a continuous, safe, and engaging environment. This approach ensures that children can experience a sense of security even beyond familiar residential settings while also enjoying a diverse and enriching environment for personal growth (Molnar et al., 2004).

From the perspective of environmental elements, optimization can be achieved by enhancing the core factors that contribute to children's sense of safety (Yang et al., 2024). For elements that positively impact children's safety perception, such as vegetation, water bodies, and sidewalks, improving their accessibility and child-friendliness should be prioritized (Villanueva et al., 2014). For vegetation, create multilayered green belts with low shrubs and colorful plants to reduce traffic distractions and spark curiosity. Water bodies can be designed as shallow, interactive fountains with non-slip paving and transparent railings, satisfying children's attraction to water while ensuring safety. Sidewalk optimization should balance width, avoiding being too narrow or too wide, and incorporate ground murals and elastic paving materials for guidance and buffering. For factors causing safety anxiety, establish a systemic intervention mechanism. Implement time and space separation in traffic management, like speed limits during school hours and variable lane designs. For billboards and signage, prioritize child sightlines by raising electronic screens and bright signs to minimize distractions. Innovate street furniture designs, such as canopy-like sunshades, can optimize skyline visibility and redirect children's attention to ground—level activities.

This study's framework is proven applicable to urban perception assessment, especially research from children's perspectives. The Photovoice method, as a key part of this framework, effectively captures authentic subjective views of children that are often overlooked in adult-led evaluations, which further validates the framework's reliability. Findings derived from this framework hold significant application value for Chinese urban contexts: they can provide guidance for targeted interventions in old communities (e.g., updating aging infrastructure to enhance child safety) and communities with dense child populations (e.g., optimizing public space design to adapt to children's activity needs), directly supporting the implementation of child-friendly city initiatives across the country. This not only confirms the transferability of the research framework in urban perception assessment but also lays a foundation for its promotion and application in more Chinese cities.

### 4.4 Limitations and future research directions

This study adopts a child-centric approach, conducting research based on children's unique physiological and psychological characteristics to achieve truly child-oriented inquiry. While it focuses on age differences and clearly identifies disparities in environmental safety perception between children and adults, several limitations persist: no efficient method for collecting child-perspective images has been developed, resulting in insufficient samples that confine the study to child-dense communities and cast doubts on its applicability to other urban environments; it relies primarily on visual indicators to assess children's perceptions, overlooking sensory factors such as sound; data were collected in December 2024 (winter) in Nanjing, a city with a subtropical monsoon climate—though the “evergreen base” of community vegetation mitigates seasonal interference, winter vegetation withering may still compromise the seasonal universality of conclusions; and it only targets children aged 7-12, failing to cover the full spectrum of children's perceptual characteristics due to unaddressed age-related variations in cognition and development.

Drawing on these limitations, future research can progress in three areas: expand the child age range to include preschool to adolescent groups, refining the mechanism by which age differences influence environmental perception; explore efficient child-perspective image collection methods to increase sample sizes and broaden the types of study areas, thereby enhancing the applicability of conclusions to diverse urban settings; and integrate multi-sensory indicators into perception assessment, while addressing seasonal bias through four-season data collection and the construction of seasonally adaptive models. These measures will provide comprehensive, scientific support for child-friendly urban planning and renovation, facilitating urban sustainable development.

## 5 Conclusion

Although many existing studies have used SVIs and deep learning methods to predict and quantify safety perception in urban environments, few have applied these methods specifically to analyze children's perceptions. Moreover, most studies have not thoroughly explored the differences between children and adults in their safety perception of environmental factors. Due to their unique physiological characteristics and underdeveloped psychological state, children's emotional responses to environmental factors often differ significantly from those of adults. Ignoring children's real experiences and needs in the urban environment would prevent the true realization of child-friendly city planning. Therefore, this study adopts a dual-perspective approach, incorporating children's and adults' viewpoints. Using deep learning techniques, elements from SVIs were extracted, besides, an artistic and creative participatory research method, “Photovoice” was used to establish a safety perception dataset. This dataset was then integrated into a machine learning model for prediction and optimization, enabling an objective evaluation of safety perception across the entire environment. Finally, a random forest regression model was applied to explore the non-linear differences in how various street environmental factors influence the safety perception of children and adults.

Regarding the differences in safety perception of street environments between children and adults, this study found significant variations in their spatial preferences, attention focus, emotional response patterns, and the influence of specific environmental factors. In terms of spatial preference, children tend to favor residential interior environments, while adults are more adaptable to external urban spaces. Regarding emotional response patterns, children's perception is more intuitive and follows a relatively linear trend, whereas adults exhibit more complex, non-linear emotional fluctuations in response to environmental changes. In terms of environmental factor influence, children show a positive safety preference for elements such as the sky, water bodies, and sidewalks, while other factors generally exert various degrees of negative influence. By contrast, adults tend to exhibit threshold effects, meaning their safety perception changes only after certain environmental factors reach specific levels. The two groups also show notable differences in how they perceive the safety impact of motor vehicles, buildings, billboards, the sky, and bicycles. Therefore, rationally planning and allocating environmental elements within the built environment is crucial to balance the safety needs of children and adults. By doing so, urban spaces can be designed to be secure and developmentally supportive, fostering an environment that promotes physical and mental wellbeing across different age groups.

## Data Availability

The raw data supporting the conclusions of this article will be made available by the authors, without undue reservation.
